# Document-level biomedical relation extraction via hierarchical tree graph and relation segmentation module

**DOI:** 10.1093/bioinformatics/btae418

**Published:** 2024-06-25

**Authors:** Jianyuan Yuan, Fengyu Zhang, Yimeng Qiu, Hongfei Lin, Yijia Zhang

**Affiliations:** School of Information Science and Technology, Dalian Maritime University, Dalian 116026, China; School of Information Science and Technology, Dalian Maritime University, Dalian 116026, China; School of Information Science and Technology, Dalian Maritime University, Dalian 116026, China; School of Computer Science and Technology, Dalian University of Technology, Dalian 116024, China; School of Information Science and Technology, Dalian Maritime University, Dalian 116026, China

## Abstract

**Motivation:**

Biomedical relation extraction at the document level (Bio-DocRE) involves extracting relation instances from biomedical texts that span multiple sentences, often containing various entity concepts such as genes, diseases, chemicals, variants, etc. Currently, this task is usually implemented based on graphs or transformers. However, most work directly models entity features to relation prediction, ignoring the effectiveness of entity pair information as an intermediate state for relation prediction. In this article, we decouple this task into a three-stage process to capture sufficient information for improving relation prediction.

**Results:**

We propose an innovative framework HTGRS for Bio-DocRE, which constructs a hierarchical tree graph (HTG) to integrate key information sources in the document, achieving relation reasoning based on entity. In addition, inspired by the idea of semantic segmentation, we conceptualize the task as a table-filling problem and develop a relation segmentation (RS) module to enhance relation reasoning based on the entity pair. Extensive experiments on three datasets show that the proposed framework outperforms the state-of-the-art methods and achieves superior performance.

**Availability and implementation:**

Our source code is available at https://github.com/passengeryjy/HTGRS.

## 1 Introduction

In the face of the increasing amount of biomedical literature, biomedical text mining techniques can efficiently analyse relevant literature information for biomedical researchers. Biomedical relation extraction is a crucial task in biomedical text mining, focusing on extracting relationships between entities from unstructured text. This process plays a vital role in various downstream applications, such as biomedical knowledge base construction (Li *et al.* 2019), information retrieval (Wang *et al.* 2017). Many excellent studies are proposed to advance the field of biomedical relation extraction (Zheng *et al.* 2017, Peng *et al.* 2018). However, previous methods usually focus primarily on sentence-level biomedical relation extraction, where subject and object entities present in a single sentence. Due to the abundance of relation instances expressed by more than one sentence in the real biomedical literature, more and more researchers are shifting their attention to document-level biomedical relation extraction (Bio-DocRE) (Verga *et al.* 2018, Jia *et al.* 2019).

For Bio-DocRE, many entities are often distributed across multiple sentences in a single document, posing a significant challenge for identifying inter-sentence relation facts. The model needs to comprehend contextual information and possess logical reasoning capabilities (Yao *et al.* 2019). Effective identification of relation instances that span across sentences has become a key research area in this task. Early Bio-DocRE models primarily relied on convolutional neural networks (CNN), recurrent neural networks (RNN), or LSTM to encode the entire document. However, these approaches were limited by the encoder’s context modeling capabilities, making it challenging to capture long-range dependencies.

One line of research in Bio-DocRE is based on transformers to tackle these challenges. Due to the excellent ability of transformer architecture to capture long-distance dependencies, several approaches have been proposed (Lai and Lu 2020, Xu *et al.* 2021, Zhou *et al.* 2021, Xiao *et al.* 2022). In particular, Zhou *et al.* (2021) introduced a localized context pooling concept based on the transformer architecture to capture long-range contextual information comprehensively. These models only use text sequences as model inputs and further incorporate the internal structure of entities within the encoder, which does not allow the model to learn the external interaction among entities explicitly. Therefore, it is difficult to extract cross-sentence relation instances that require reasoning (Zhang *et al.* 2023).

Another line of research in Bio-DocRE direction involves implicitly modeling information interactions between entities to improve the model’s inference capabilities. This approach aims to address the reasoning limitations observed in transformer-based methods by drawing inspiration from graph neural networks (GNNs) (Zhang *et al.* 2019). Researchers have proposed numerous works based on graph structures to model logical inference, called graph-based models or GNN-based models. To better identify inter-sentence relations, unlike directly using transformer structure to model the task, recent graph-based methods (Nan *et al.* 2020, Tran *et al.* 2020, Wang *et al.* 2020) transform the text sequences into graphs, where the nodes represent entities or mentions. Then, a GNN will be leveraged to capture the information interaction among entities in the document graph.

**Figure 1.**
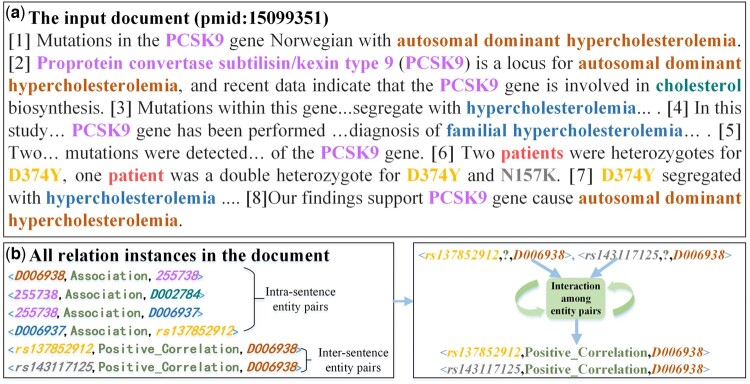
Overview of a motivation example document from BioRED dataset. (a) An input document, where each entity’s mention is labeled with the same color; (b) all relation instances where each entity and its corresponding concept identifier IDs have the same color. The lower right part shows two inter-sentence relation instances which require reasoning techniques to be identified.

The aforementioned methods have all, to varying extents, improved the model’s ability to extract inter-sentence relation instances by capturing long-range dependencies or modeling interactive reasoning between entities. However, much of the previous research primarily models the Bio-DocRE task by capturing entity features directly to relation prediction, with limited consideration for the effectiveness of entity pair information in the intermediate step. As displayed in Fig. 1, it is easy for previous methods to identify the relation type of intra-sentence entity pairs, such as (*D006938*, *255738*) and (*D006937*, *rs137852912*), through capturing the entity features. However, for a model that directly identifies the relations after obtaining entity features, it is not easy to correctly predict inter-sentence relation instances, such as (*rs137852912*, *D006938*) and (*rs143117125*, *D006938*), of which prediction depend on the interaction with other entity pairs.

To mitigate the model’s underutilization of entity pair information, we decouple the Bio-DocRE task into three phases, with the first phase modeling entity-level interaction reasoning, the second phase modeling entity pair level interaction reasoning, and the third phase relation classification. In the first stage, we reconstruct document sequences as hierarchical tree graphs (HTGs) with structural dependencies to obtain more expressive entity features. It is worth noting that the current graph-based approach lacks the modeling of entity context information; they usually construct document graphs based on entity or mention granularity, and the connecting edges between nodes do not reflect hierarchical relationships. Considering that entities are highly correlated with their local context information, which in most cases can reflect some feature information of entities (Huang *et al.* 2021); we achieve fine-grained in-depth modeling of document sequences by introducing document node and local context nodes.

The interaction among entity pairs can significantly facilitate relation extraction. To this end, we redefine Bio-DocRE task as a table-filling task (Miwa and Sasaki 2014) and propose a relation segmentation (RS) module. Specifically, we draw inspiration from convolutional networks used in computer vision tasks. We first map the relevant features of entity pairs into a feature matrix, where each cell represents the feature information of the corresponding entity pair. Then, we update the feature in the matrix using RS module. Additionally, we leverage criss-cross attention, inspired by Huang *et al.* (2019), to enhance the depth of interaction between entity pairs during reasoning. To validate the effectiveness of our model, extensive experiments on three widely used Bio-DocRE datasets and our approach outperforms recent SOTA. To summarize, in this article, we decouple the Bio-DocRE task into three stages so that the model captures global document information from intermediate steps. This article’s contributions can be outlined as:

- We propose the model HTGRS by decomposing Bio-DocRE task into three stages. This decoupling allows the model to conduct global interaction reasoning better by focusing on entities and entity pairs.
- In the first stage, we reconstruct the document sequence into an HTG, through finely joint modeling of different hierarchical information in the document to capture more expressive entity features. In the second stage, we treat Bio-DocRE as a table-filling task and propose a RS module to improve the model’s inference capability through global interaction among entity pairs.
- The proposed model undergoes evaluation using three benchmark datasets in the biomedical field. Extensive experimental results demonstrate that HTGRS has a significant improvement over current state-of-the-art (SOTA) methods.

## 2 Related work

Sentence-level biomedical relation extraction methods face challenges in recognizing relation instances that necessitate cross-sentence reasoning, and they struggle to address document-level task scenarios effectively. Consequently, document-level biomedical relation extraction has gained prominence as a research focus, gradually superseding sentence-level biomedical relation extraction. This section will discuss the current mainstream works in Bio-DocRE.

### 2.1 Graph-based methods

GNNs are well-suited for modeling long-distance dependencies among entities in biomedical documents and demonstrate strong reasoning capabilities; many researchers leverage GNNs to advance Bio-DocRE better (Christopoulou *et al.* 2019, Nan *et al.* 2020, Tran *et al.* 2020, Wang *et al.* 2020). Typically, these methods construct a document graph using mentions, entities, and sentences as nodes. The edges are then defined based on heuristic rules or semantic considerations, and the GNNs are exploited to conduct implicit reasoning on the document graph. For example, Wang *et al.* (2020) build a document graph containing three nodes: mentions, entities, and sentences, and propose a global-to-local mechanism to aggregate the structural information of entities. Nan *et al.* (2020) introduce a novel model called LSR to fully utilize the reasoning capabilities of GNNs. In their approach, they regard the document graph as a latent variable and dynamically update the graph structure by incorporating attention mechanisms into GNNs.

### 2.2 Transformer-based methods

Simultaneously, pre-trained language models based on the transformer architecture have gained considerable attention in the field of natural language processing. Because of the transformer’s advantage in modeling long-distance dependency relationships, researchers have proposed numerous transformer-based methods (Xu *et al.* 2021, Zhou *et al.* 2021, Xiao *et al.* 2022), directly employing pre-trained language models to extract intra-sentence and inter-sentence relation instances in biomedical documents. Xu *et al.* (2021) propose the SSAN model that incorporates prior structural knowledge of entities into the self-attention layers of the transformer and the knowledge is propagated throughout the entire encoding phase. Furthermore, Zhou *et al.* (2021) propose the ATLOP model with two contributions: adaptive thresholding and local context pooling.

Certain studies, whether graph-based or transformer-based, have made progress in addressing specific challenges of Bio-DocRE, such as long-range dependencies and reasoning ability for complex inter-sentence instances. However, these methods primarily focus on entity-level information, often proceeding to relation prediction directly after obtaining entity features. They overlook the effectiveness of interactions between entity pairs as an intermediate layer for relation extraction. The HTGRS proposed in this paper surpasses them in two aspects: (1) In the first stage, we construct an HTG that facilitates fine-grained deep modeling of crucial information in the document, resulting in more expressive entity features. (2) In the second stage, treating relevant entity pair features as an image, we propose a RS module to better model the relation reasoning based on entity pairs.

## 3 Materials and methods

**Figure 2.**
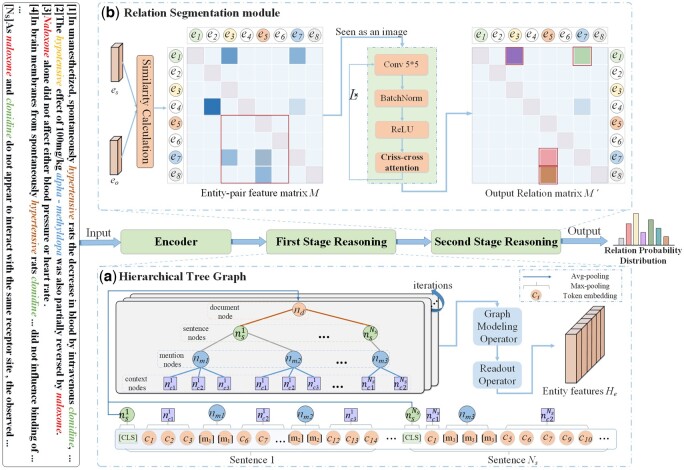
An overview of the proposed HTGRS for Bio-DocRE. The biomedical document is fed into the encoder to produce token embeddings. (a) After encoding, we obtain the feature of each node by pooling operator and feed the HTG into R-GCNs to conduct first-stage reasoning. The graph modeling operator and readout operator denote the node information propagation and feature pooling of mention nodes, respectively. (b) Constructing entity pair feature matrix by the entity features from first-stage reasoning and we propose a relation segmentation module to capture local and global interaction dependency information between entity pairs.

**Figure 3.**
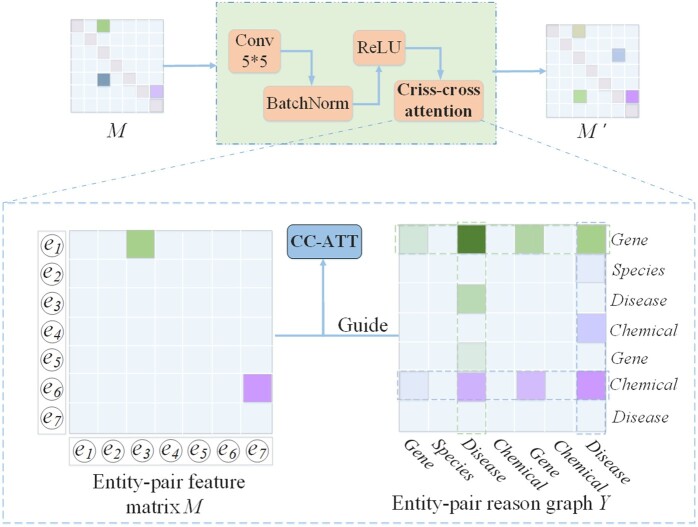
The details of criss-cross attention in RS module.

In this section, the implementation details of our proposed HTGRS framework are introduced. Figure 2 shows the HTGRS architecture and Fig. 3 illustrates the details of RS. Based on three stages for Bio-DocRE, we first reconstruct the input document into an HTG to model relation reasoning based on entities (Section 3.2). Then, we convert Bio-DocRE into a table-filling task to model entity pairs-level relation reasoning (Section 3.3).

### 3.1 Problem formulation

With a biomedical document *D* consists of entities $E_{D}={\left\{e_{i}\right\}}_{i}^{N}$, sentences $S_{D}={\left\{s_{i}\right\}}_{i}^{\left|N_{s}\right|}$, and tokens $T_{D}={\left\{t_{i}\right\}}_{i}^{\left|N_{t}\right|}$, Bio-DocRE task aims to identify the specific relation r∈R for an entity pair. In the formulation, we do not directly conduct relation prediction between the head entity *e_s_* and tail entity *e_o_* after capturing the entity features. To model interactive reasoning among entity pairs, we construct a *N *×* N* entity pair feature matrix *M*, where *N* denotes the maximum number of entities in all samples and each cell $M_{s,o}\in M$ indicates the feature representation between *e_s_* and *e_o_*. Note that the constructed entity pair feature matrix, matrix cells, and relation categories are aligned with the image, pixel, and pixel-affiliated semantic labels in semantic segmentation, respectively.

### 3.2 Relation reasoning based on entities

#### 3.2.1 Document encoding

For an input document $D={\left\{t_{i}\right\}}_{i}^{L}$, where *L* denotes the total token lengths, we utilize a biomedical pre-trained language model as the encoder to obtain the initial embedding of each token and we insert token “[CLS]” and “[SEP]” at the begin and end of each sentence *s*. Additionally, to account for the fact that an entity may have multiple mentions within a document, we incorporate a special token “&” before and after each mention to serve as an entity marker (Verga *et al.* 2018). The embedding of marker “&” placed at the beginning position serves as the representation for the mention. After encoding, the embeddings of each token are as follows:


(1) $$H=\left[h_{1},h_{2},\ldots ,h_{L}\right]=\mathrm{PLM}\left(\left[t_{1},t_{2},\ldots ,t_{L}\right]\right),$$

where $H\in R^{L\times d_{\mathrm{emb}}}$. *h_i_* is the embedding of each token, we obtain the embedding representation $n_{m_{i}}\in R^{1\times d_{\mathrm{emb}}}$ of mentions through special tokens “&”.

#### 3.2.2 HTG construction

To model first-stage relation reasoning based on entity-level information, we transfer the input document sequence into an HTG G=(V,E), which is constructed by considering multi-dimensional textual information. In general, a biomedical document contains four hierarchical levels of direct elemental information: global document features, sentence features, mention features, and local context features. Previous graph-based approaches in constructing heterogeneous graphs mostly focused on mention or entity dimensions. This approach undoubtedly results in the loss of hierarchical dependency information within a given document. Inspired by Huang *et al.* (2021), it is essential to consider the local context information of mentions for obtaining expressive entity features. To be specific, we define four types of nodes: document, sentence, mention, and local context, which covers key elements of various hierarchies in the text. To distinguish structure dependencies between nodes of different hierarchies, three edges are defined: D–S edges (document, sentence), S–M edges (sentence, mention), and M–C edges (mention, local context).

After encoding the document, we model the nodes in the hierarchical graph using the embedding vectors of the text sequence tokens *H*. For the sentence node, we utilize the token [CLS] located at the beginning of each sentence as the node representation $n_{s_{i}}\in R^{1\times d_{\mathrm{emb}}}$. Then, we obtain the feature representation $n_{d}\in R^{1\times d_{\mathrm{emb}}}$ of document nodes by averaging the embeddings of all sentences belonging to the same document. When an entity mention exists in a sentence, the sentence is segmented into several context regions around the mention. The embeddings of each sentence are as follows: $S_{i}=\{c_{1},c_{2},c_{3},\ldots ,c_{n}\}$, where *n* represents the number of tokens after word segmentation processing and *c_n_* denotes the embedding of each token. The token embeddings adjacent to the mention embedding are then treated as corresponding local context regions. As shown in Fig. 2, sentence 1 contains $\mathrm{mentio}n_{1}=\{c_{4},c_{5}\}$ and $\mathrm{mentio}n_{2}=\{c_{10},c_{11}\}$, the token embedding of this sentence are divided into three local context areas by these mention tokens, $\mathrm{contex}t_{1}^{1}=\{c_{1},c_{2},c_{3}\},\;\mathrm{contex}t_{2}^{1}=\{c_{6},c_{7},\ldots \},\;\mathrm{contex}t_{3}^{1}=\{c_{12},c_{13},c_{14}\}$ serve as local context information for these two mentions. Then, we obtain the local context node features $n_{c_{i}}^{s}\in R^{1\times d_{\mathrm{emb}}},s\in \left|N_{s}\right|$ through max-pooling operation of these local context token embeddings. It is noteworthy that, for sentences where no mentions occur, we do not retain their information in the construction of the HTG to reduce the impact of irrelevant information.

When the HTG is constructed, we utilize the relational graph convolutional network (R-GCN) (Schlichtkrull *et al.* 2018) to model the interaction reasoning based on entity-level. The process of information propagation and feature updating between nodes at different levels is as follows:


(2) $$h_{n}^{l+1}=\sigma \left(\sum_{r\in R}\sum_{u\in N_{n}^{r}}\frac{1}{c_{nr}}W_{r}^{\left(l\right)}h_{u}^{\left(l\right)}+W_{o}^{\left(l\right)}h_{n}^{\left(l\right)}\right),$$

where σ(·) denotes activation function, which ReLU is utilized in our model. $N_{n}^{r}$ represents the set of neighbor nodes of node *n* with edge type r∈R, *c_nr_* is a regularization constant for specific problem (we set $c_{nr}=|N_{n}^{r}|$). $W_{r}^{l},\;W_{o}^{l}$ are trainable weight parameters. Furthermore, due to the nodes at different layers of R-GCN have varying interactive information expressive capabilities for the HTG, we concatenate the hidden state vectors of each layer to obtain the final feature representation *h_n_* of node *n*:


(3) $$h_{n}=\left[h_{n}^{\left(0\right)};h_{n}^{\left(1\right)};\ldots ;h_{n}^{\left(L\right)}\right].$$

### 3.3 Relation reasoning based on entity pairs

Since entity pairs serve as effective indirect representations of relations between entities, after the first-stage reasoning, we treat the Bio-DocRE problem as a table-filling task to model relation reasoning based on entity pairs. RS module is proposed inspired by the U-net (Ronneberger *et al.* 2015). Specifically, we initially employ the logsumexp pooling (Jia *et al.* 2019) to obtain the embedding of each entity *e_i_* from corresponding mention node. Formally, the entity representation *e_i_* is computed as follows:


(4) $$e_{i}=\log\sum_{j=1}^{N_{e_{i}}}exp\left(m_{ij}\right),$$

where *m_ij_* denotes the mention node representation from HTG and $N_{e_{i}}$ represents the total number of mentions belonging to the same entity.

Then, we calculate the **relation matrix**  $M\in R^{N_{e}\times N_{e}\times D}$ to model the relation reasoning based on entity pairs. Each cell in the matrix *M* represents an embedding vector $M_{so}\in R^{1\times d_{\mathrm{emb}}}$, which is the feature representation of entity pair $\left(e_{s},e_{o}\right)$. *M_so_* is computed through element-wise multiplication as follows:


(5) $$M_{so}=[e_{s}⊙e_{o}],M_{so}\in R^{1\times D},$$

where ⊙ denotes the element-wise similarity.

Figure 2b illustrates that the RS module consists of *L* reasoning layers and each reasoning layer is composed of four components: a 2D convolution layer with kernel 5 × 5, a BatchNorm layer, a ReLU layer, and a criss-cross attention. Regarding the relation matrix *M* as an image with *D* channels, where the height and width are denoted as *N*. *D* is the embedding dimension and *N* denotes the maximum number of entities from all samples. Relation matrix *M* is updated as follow:


(6) $$M^{\prime }=CCA_{M}^{L}(\mathrm{Con}v_{M}^{L}(W_{M}M)),M^{\prime }\in R^{N\times N\times D},$$

where $\mathrm{Con}v_{M}^{L}$ and $CCA_{M}^{L}$ denote the *L* convolutional network and criss-cross attention. *W_M_* is the learnable weight matrix.

The relation prediction of an entity pair is largely affected by its overlapping entity pairs. For example, the entity pair $\left(e_{1},e_{3}\right)$ in Fig. 3 can focus on the features of entity pairs $\left(e_{1},e_{*}\right)$ and $\left(e_{*},e_{3}\right)$ in its horizontal and vertical directions. Hence, the feature of cell *M_so_* is updated after *Conv* to achieve deeper interaction reasoning between entity pairs:


(7) $$M_{so}=\sum_{i=1}^{N_{e}}\left(A_{\left(s,o\right)\to (s,i)}M_{si}+A_{\left(s,o\right)\to (i,o)}M_{io}\right),$$

where $A_{\left(s,o\right)\to (s,i)}$ and $A_{\left(s,o\right)\to (i,o)}$ denote the attention of *M_so_* to the features of other entity pairs in row and column.

### 3.4 Relation classification

In the third stage, we use a bilinear function for relation classification. Specifically, given the entity features *e_s_* and *e_o_* obtained from the first-stage reasoning and the entity pair embedding *M_so_*, we then map them to final hidden vectors for relation classification using FFNN. Subsequently, the probability of the relation to which the entity pair belongs is calculated using a bilinear function. The calculation process is as follows:


(8) $$z_{s}=\tanh\left(W_{s}e_{s}+M_{so}\right),$$

(9) $$z_{o}=\tanh\left(W_{o}e_{o}+M_{so}\right),$$

(10) $$P\left(r|{\tilde{e}}_{s},{\tilde{e}}_{o}\right)=\sigma \left(z_{s}W_{r}z_{o}+b_{r}\right),$$

where $W_{s}\in R^{d_{\mathrm{emb}}\times d_{\mathrm{emb}}},\;W_{o}\in R^{d_{\mathrm{emb}}\times d_{\mathrm{emb}}}W_{r}\in R^{d_{\mathrm{emb}}\times d_{\mathrm{emb}}}$, and $b_{r}\in R$ are learnable parameters.

### 3.5 Training objectives

To train the proposed model HTGRS, we follow Zhou *et al.* (2021) by adopting an adaptive threshold loss to optimize the training objective for Bio-DocRE as follows:


(11) $$L_{1}=-\sum_{r\in P_{T}}\;\text{log}\;\left(\frac{\;\text{exp}\;\left(\mathrm{logi}t_{r}\right)}{\sum_{r\prime \in P_{T}\cup \left\{TH\right\}}\;\text{exp}\;\left(\mathrm{logi}t_{r\prime }\right)}\right),$$

(12) $$L_{2}=-\text{log}\;\left(\frac{\;\text{exp}\;\left(\mathrm{logi}t_{TH}\right)}{\sum_{r\prime \in N_{T}\cup \left\{TH\right\}}\;\text{exp}\;\left(\mathrm{logi}t_{r\prime }\right)}\right),$$

(13) $$L_{\mathrm{final}}=L_{1}+L_{2},$$

where *P_T_* and *N_T_* represent the positive class and negative class, respectively. *TH* is an introduced dynamic threshold class. The goal is to make the logits of positive class tend to be higher than the threshold class *TH*, and those of negative class tend to be lower than the threshold class *TH*.

## 4 Experiments results

### 4.1 Datasets and evaluation

We comprehensively evaluate the proposed model using three Bio-DocRE datasets: CDR, GDA, and BioRED. Specifically, CDR (Li *et al.* 2016) is published by the BioCreative-V community, which contains 1500 PubMed abstracts. GDA (Wu *et al.* 2019) contains 30 192 PubMed abstracts with diseases and genes entity. BioRED (Luo *et al.* 2022) is a recently introduced Bio-DocRE dataset that features diverse entity types and relation categories. It comprises six entity concept types and eight common relation types. Table 1 provides further details about these datasets. The implementation details are listed in Supplementary Material.

**Table 1.** Statistics of three document-level biomedical relation extraction datasets.

Statistics	CDR	GDA	BioRED
Train set	500	23 353	400
Dev set	500	5839	100
Test set	500	1000	100
Number of Rels^a^	3116	46 343	6503
Number of inter Rels^b^	832	7463	–
Avg sentences per Doc	9.7	10.2	11.9
Avg entities per Doc	7.6	5.4	3.8

a Number of relation instances.

b Number of inter-sentence relation instances.

This article employs evaluation metrics consistent with official standards to reasonably assess the performance of proposed model on Bio-DocRE tasks, including precision (*P*), recall (*R*), and *F*1 score and specificity (TNR). Furthermore, we report the intra-*F*1 and inter-*F*1 scores of model, which evaluate the extraction effect for intra-sentence and inter-sentence relation instances, respectively.

### 4.2 Compared methods

Since the BioRED dataset is a novel dataset for Bio-DocRE, baseline models need to be compared separately. We compare the proposed model with the following SOTA methods on CDR and GDA datasets:

- Recent Bio-DocRE methods mainly include the benchmark of BERT-GT (Lai and Lu 2020), LSR (Nan *et al.* 2020), EoGANE (Tran *et al.* 2020), SSAN (Xu *et al.* 2021), B-KGAGN (Sun *et al.* 2023), seq2seq (Giorgi *et al.* 2022), SAIS (Xiao *et al.* 2022), SD-KD (Zhang *et al.* 2023).

For BioRED dataset, we adhere to the setup of Luo *et al.* (2022), utilizing BERT-GT (Lai and Lu 2020) and PubMedBERT (Gu *et al.* 2021) as comparison models. Furthermore, to ensure comprehensive evaluation, we independently reproduce three robust baselines: SSAN (Xu *et al.* 2021), ATLOP (Zhou *et al.* 2021), SAIS (Xiao *et al.* 2022) for this dataset.

### 4.3 Main results

Tables 2 and 3 report the results of HTGRS methods in three benchmark datasets. Specifically, Table 2 shows that: (i) the proposed HTGRS obtains the best performance, demonstrating its effectiveness. The *F*1 score on CDR and GDA are 86.9% and 87.3%, respectively. (ii) Compared with the SOTA model SAIS (Xiao *et al.* 2022) among all the baselines, our model achieves up to 8.2% *F*1 improvement on CDR. (iii) Compared with SAIS, a method that explicitly guides the model to capture relevant context and entity types, we can see that the improvement in the *F*1 score on the GDA dataset is not very significant, only increasing by 0.5%. We speculate that this is due to the training scale of the GDA dataset being nearly 50 times larger than the CDR dataset, which reduces its sensitivity to the model. This observation is supported by the performance of many baseline models in terms of *F*1 scores. Comparing the TNR metric, HTGRS outperforms SAIS by 1.9%, indicating that our model is superior to the latest SOTA model. (iv) It shows that decoupling Bio-DocRE into three stages can effectively enhance model’s ability to capture of entity-level and entity pair level feature information, leading to better relation prediction. In Table 3, we observe that: (v) our method outperforms all baselines and also achieves the SOTA performance in terms of *F*1 scores on BioRED dataset, demonstrating effectiveness of the proposed model. (vi) Compared with SAIS, our model achieves a 3.1% higher *F*1 score. It is shown that the proposed model still performs well in multi-relation category scenarios through deep interactive reasoning modeling.

**Table 2.** Main experiment results on CDR and GDA datasets.^a^

	CDR				GDA			
Model	*P* (%)	*R* (%)	*F*1 (%)	TNR (%)	*P* (%)	*R* (%)	*F*1 (%)	TNR (%)
BERT-GT (Lai and Lu 2020)	64.9	67.1	66.0	–	–	–	–	–
LSR (Nan *et al.*^ 2020^)	$\tilde{64.2}$	$\tilde{65.4}$	$\tilde{64.8}$	$\tilde{65.1}$	$\tilde{80.3}$	$\tilde{84.2}$	$\tilde{82.2}$	$\tilde{78.5}$
EoGANE (Tran *et al.*^ 2020^)	–	–	66.1	–	–	–	82.8	–
SSAN (Xu *et al.*^ 2021^)	$\tilde{65.5}$	$\tilde{72.2}$	$\tilde{68.7}$	$\tilde{71.2}$	$\tilde{78.8}$	** $\tilde{89.2}$ **	$\tilde{83.7}$	$\tilde{82.7}$
ATLOP (Zhou *et al.*^ 2021^)	$\tilde{67.0}$	$\tilde{73.1}$	$\tilde{69.9}$	$\tilde{88.9}$	$\tilde{80.6}$	$\tilde{87.6}$	$\tilde{84.0}$	$\tilde{87.4}$
B-KGAGN (Sun *et al.*^ 2023^)	72.7	73.0	72.9	–	–	–	–	–
seq2seq (Giorgi *et al.*^ 2022^)	68.2	66.2	67.2	–	84.4	85.3	84.9	–
SAIS (Xiao *et al.*^ 2022^)	$\underline{\tilde{75.4}}$	$\underline{\tilde{82.3}}$	$\underline{\tilde{78.7}}$	$\underline{\tilde{93.1}}$	$\underline{\tilde{84.7}}$	$\underline{\tilde{89.0}}$	$\underline{\tilde{86.8}}$	$\underline{\tilde{89.2}}$
SD-KD (Zhang *et al.*^ 2023^)	–	–	76.8	–	–	–	86.4	–
HTGRS (ours)	**84.4**	**89.4**	**86.9**	**95.6**	**86.1**	88.6	**87.3**	**91.1**

a The ˜ denotes the results are reproduced in our setting. All the test performances are derived from the best checkpoint of the dev set. The bold values denote the highest scores and the second-best score is $\underline{\mathrm{underlined}}$.

**Table 3.** Main experiment results on BioRED dataset.^a^

	BioRED		
Model	*P* (%)	*R* (%)	*F*1 (%)
BERT-GT (Lai and Lu 2020)	$\tilde{55.0}$	$\tilde{58.7}$	$\tilde{56.8}$
PubMedBERT (Gu *et al.*^ 2021^)	$\tilde{54.2}$	$\tilde{63.8}$	$\tilde{58.6}$
SSAN (Xu *et al.*^ 2021^)	$\tilde{54.9}$	$\underline{\tilde{69.2}}$	$\tilde{61.2}$
ATLOP (Zhou *et al.*^ 2021^)	$\tilde{58.7}$	$\tilde{68.4}$	$\tilde{63.1}$
SAIS (Xiao *et al.*^ 2022^)	$\tilde{60.5}$	$\tilde{67.1}$	$\underline{\tilde{63.8}}$
HTGRS (ours)	$\underline{59.3}$	**76.8**	**66.9**

a The ˜ denotes the results are reproduced in our setting. All the test performances are derived from the best checkpoint of the dev set. The bold values denote the highest scores and the second-best score is $\underline{\mathrm{underlined}}$.

## 5 Discussion

### 5.1 Ablation studies

In Table 4, we study the influence of the HTG and RS module on three datasets through ablation studies. The w/o denotes that the module is removed in the training stage. It is evident that the model without the HTG or RS module experiences a significant decrease in performance. Notably, the absence of the RS module leads to a more pronounced decline, resulting in reductions in *F*1 score of 17.4%, 3.9%, and 6.4% for the CDR, GDA, and BioRED datasets, respectively. Furthermore, while disabling the HTG results in a decrement in performance, HTGRS still exhibits a commendable level of competence. This indicates that the global interdependence information captured by the RS module we propose is effective for document-level biomedical relation extraction. We further conduct fine-grained evaluation of the impact of RS module (see our Supplementary Material).

**Table 4.** The effects of each component on different datasets.

	CDR			GDA			BioRED		
Model	*P* (%)	*R* (%)	*F*1 (%)	*P* (%)	*R* (%)	*F*1 (%)	*P* (%)	*R* (%)	*F*1 (%)
HTGRS	84.4	89.4	86.9	86.1	88.6	87.3	59.3	76.8	66.9
*w/o* HTG	80.1	88.4	84.3	82.5	90.2	86.2	57.2	73.2	64.1
*w/o* RS	62.4	78.1	69.5	79.6	87.6	83.4	59.4	61.6	60.5

### 5.2 Performance analysis of inter/intra-sentence RE

**Figure 4.**
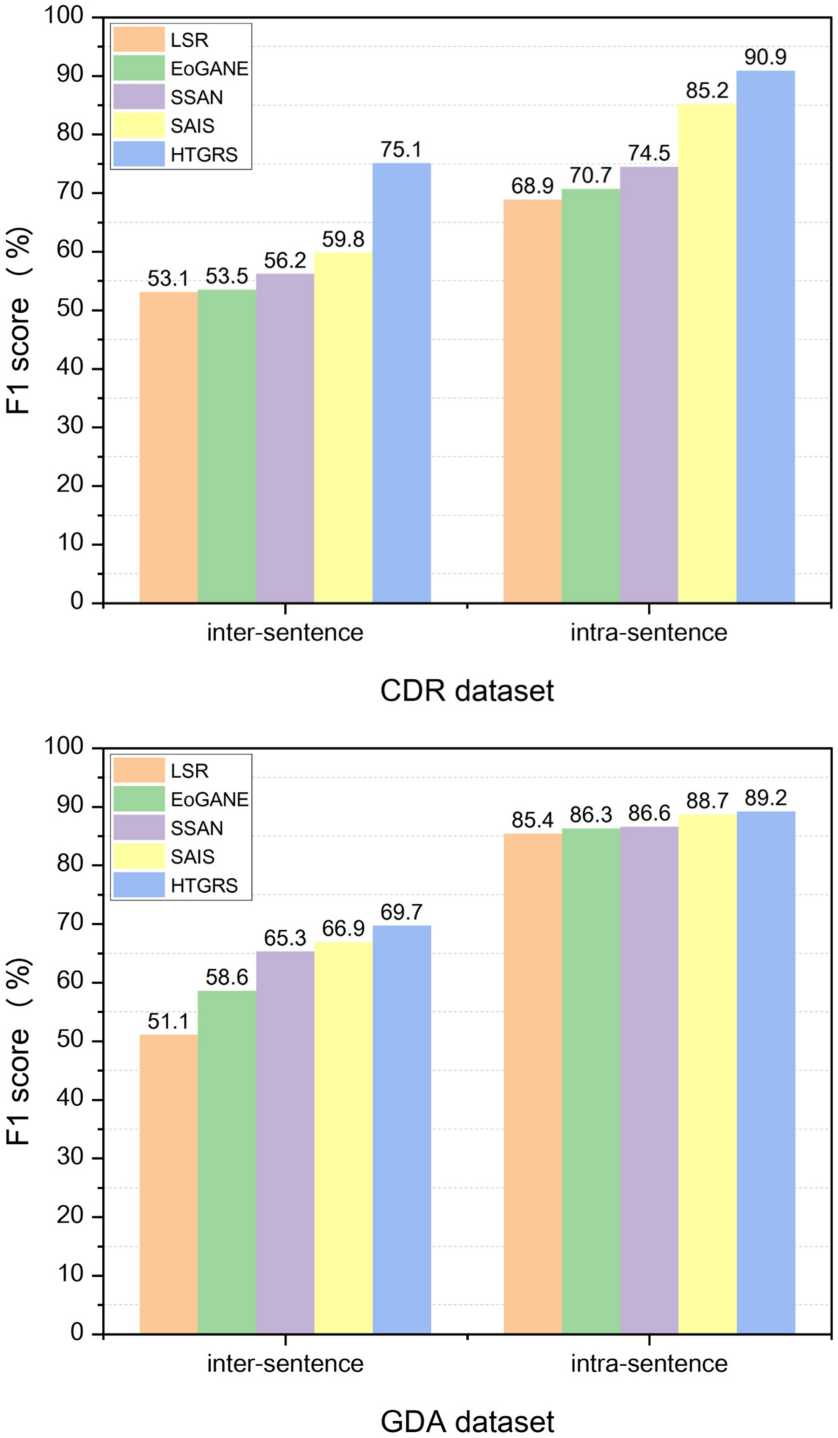
Performance of inter/intra-sentence RE. Intra-*F*1 measures the performance on the intra-sentence relation instances and inter-*F*1 evaluates the inter-sentence relation instances where none of their proper-noun mentions co-occurs.

The most significant difference between sentence-level BioRE and Bio-DocRE is that many relation instances are jointly expressed through multiple sentences. As shown in Fig. 4, we report the inter-*F*1 and intra-*F*1 of HTGRS compared with four strong baselines. We observe that HTGRS achieves a strong competitive inter-*F*1 and intra-*F*1 score on both benchmark datasets, reaching 75.1% inter-*F*1, 90.9% intra-*f*1 and 69.7% inter-*F*1, 89.2% intra-*f*1 on CDR and GDA datasets, respectively. Compared to the recent SOTA model SAIS (Xiao *et al.* 2022) with up to 15.3% and 2.8% inter-*F*1 substantial improvement on CDR and GDA, respectively, indicating that HTGRS mainly improves the performance of inter-sentence relation instances. We believe that the proposed RS module strengthens the deep interactive reasoning modeling of model based on entity pair information, thereby improving the complete capture of global document information. Furthermore, we also assess the model’s capability in extracting relation instances between entity pairs at varying distances (see our Supplementary Material).

## 6 Conclusion

In the biomedical document, multiple entities are expressed in various mention forms across multiple sentences to convey multiple biomedical relations. Most previous methods directly model features from entities to relation prediction without adequately considering the effectiveness of entity pair information for relation prediction. To overcome the limitation, we propose an HTGRS framework by decoupling Bio-DocRE into three stages, which makes the model conduct the relation reasoning based on entities and entity pairs. By constructing an HTG, HTGRS can capture contextual information more effectively. The proposed RS module allows HTGRS to fully model interactive reasoning between entity pairs. Experimental results show that the proposed HTGRS framework outperforms recent SOTA models. In the future, we will extend our method to other biomedical classification tasks, such as document-level biomedical event extraction. In addition, we also found that HTGRS has certain limitations in identifying relation instances that require background knowledge guidance. The reasonable introduction of external knowledge is also part of our future research direction.

## Supplementary Material

btae418_Supplementary_Data
