# Supplementary material for: Document-level biomedical relation extraction via hierarchical tree graph and relation segmentation module
Source: Bioinformatics. 2024 Jun 25;40(7):btae418. doi: 10.1093/bioinformatics/btae418 (PMC11629692; doi:10.1093/bioinformatics/btae418)
Supplement: btae418_Supplementary_Data [file btae418_supplementary_data.pdf]

Supplementary data for

# Document-level biomedical relation extraction via hierarchical tree graph and relation segmentation module

**Jianyuan Yuan,[1] Fengyu Zhang,[1] Yimeng Qiu,[1] Hongfei Lin[2] and Yijia Zhang [1,*]**

1 School of Information Science and Technology, Dalian Maritime University, Dalian, 116026, China and 2 School of Computer Science and Technology, Dalian University of Technology, Dalian, 116024, China

* Corresponding author. zhangyijia@dlmu.edu.cn

Availability and implementation: Our code and supplementary data are available at https://github.com/passengeryjy/HTGRS.

Supplementary information: Supplementary data are available at Bioinformatics online.

## A Implementation Details

### Experimental setup.

Our model is implemented using the PyTorch and trained on a single GTX 3080 GPU, utilizing SciBert-base as the base encoder. We set the learning rate for all experiments to 3e-5 with a warmup ratio of 0.06. The number of iterations for the hierarchical graph is set to 3, and the output dimension is set to 512, consistent with the embedding dimension. RS module has 3 iterations and an output dimension of 512. The entire model is optimized using AdamW.

## B Discussion

### Impact of hierarchical concept in document graph.

To evaluate the hierarchical concept introduced compared to the traditional document graph, we conduct a series of ablation studies by deleting the different level nodes on the CDR and BioRED dev set. The results are shown in Table S1. Specifically, when we remove the document node from the hierarchical tree graph, we observe that the model's performance drops to varying degrees on both the CDR and BioRED datasets, with drops of 1.2% and 1.1%, respectively. When we delete the sentence nodes and make the document node connect to the mention nodes, it can be seen that the performance only have a slight drop. It is worth noting that the mention information is the most direct source of entity concept features in the document, so we do not remove it from the hierarchical tree graph.

Finally, we pruned the introduced local context nodes, and the model's F1 scores dropped by 0.8% and 1.0% on the CDR and BioRED datasets, respectively. The above results show that the influence of document, local context, and sentence nodes gradually weakens the model performance. We attribute this to the fact that document node representation, as a global feature,

contributes significantly to model's ability to relation reasoning based on entity level. Since mentions and local context information are fine-grained models as crucial elements, redundant sentence information is filtered out to a certain extent. In conclusion, our findings support the notion that introducing the concept of hierarchy in document graphs is a valuable approach for modeling entity-level relation reasoning.

Table S1. The impact of node information on CDR and BioRED dev sets.

| Model | CDR | | | BioRED | | |
|---|---|---|---|---|---|---|
| | P(%) | R(%) | F1(%) | P(%) | R(%) | F1(%) |
| HTGRS | **84.4** | **89.4** | **86.9** | **59.3** | **76.8** | **66.9** |
| w/o doc_node | 84.2 | 87.3 | 85.7 | 60.1 | 72.7 | 65.8 |
| w/o sen_node | 84.2 | 88.9 | 86.5 | 58.2 | 77.0 | 66.3 |
| w/o con_node | 81.3 | 89.3 | 86.1 | 62.9 | 69.3 | 65.9 |

## Effectiveness analysis of three stage decoupling.

To further verify the effectiveness of three-stage decoupling, we assess the model HTGRS with RS module or without RS module on different groups of dev set in CDR and BioRED, respectively, which are classified into four groups based on the number of entities. Specifically, we use two steps, 5 and 7, to group CDR and BioRED based on the characteristics of the distribution of entities in the document in the dataset, respectively. Table S2 represents the group status.

As shown in Figure S1, the model's performance HTGRS or without RS module sharp drops with increasing entity numbers. As the number of entities in the document increases, the number of entity pairs involved increases rapidly, accompanied by a sharp increase in the complexity of entity pair interaction. We observe that the model with RS module consistently outperforms the model without RS module. When the number of entities increases, the performance of the model without RS module sharply declines, but the model with RS module is the opposite, declining steadily. This result demonstrates that attention to the interactive reasoning between entity pairs is useful for relation prediction, and our relation segmentation module is helpful for the model to conduct interaction reasoning.

Table S2. Proportions of samples in four groups to all dev set.

| Datasets | Four groups | | | |
|---|---|---|---|---|
| | [1,5)/ [1,7) | [5,10)/ [7,14) | [10,15)/ [14,21) | [15,-)/ [21,-) |
| CDR | 24.8% | 60.6% | 12.2% | 2.4% |
| BioRED | 15% | 49% | 21% | 15% |

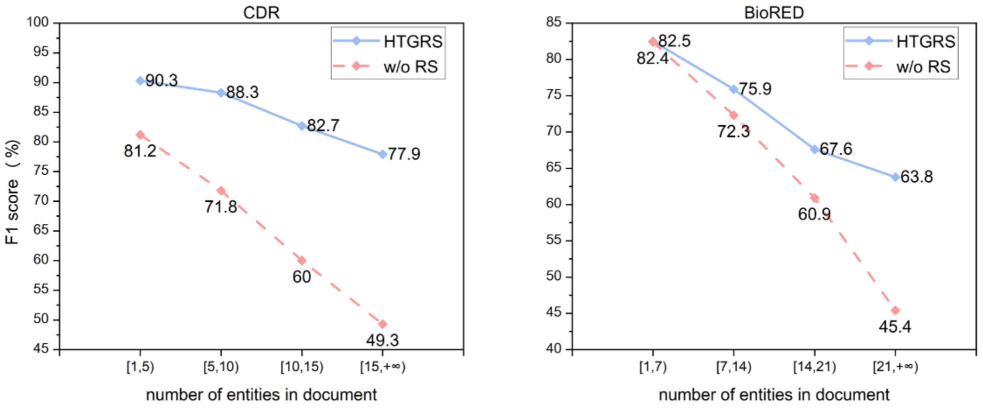


Figure S1. The effects of entity numbers on different datasets.

## Impact of entity distance.

Previous studies[1] have already shown that extracting cross-sentence relation instances has always been one of the challenges in document-level biomedical relation extraction task. In order to further explore the impact of entity distance on relation extraction (RE), we divide the development set into five subsets according to the entity distance (referring to the relative distance between the first mention of two target entities in the document) to assess model performance, and report the F1 scores of the model on the CDR and GDA datasets in Figure S2.

From Figure S2, we observe that the model HTGRS consistently outperforms both the model w/o (without) RS module and the model w/o HTG module. We notice that when the distance between the head and tail entities is greater than or equal to 64, the improvement becomes larger. In addition, the variant w/o HTG significantly outperforms the variant w/o RS module. This indicates that the RS module can effectively capture the global interaction information among the entity pairs in one document, thereby significantly improving the performance of Bio-DocRE.

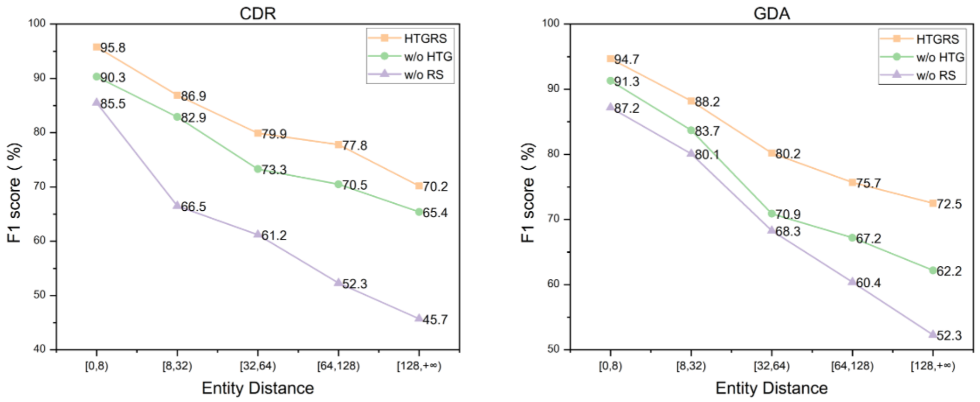


Figure S2. The effects of entity distance on different datasets.

## Case study

In addition, we randomly chose some prediction samples on the CDR dataset to analyze our emodel HTGRS further. Specifically, we select two of the most striking examples, represented in Table S3. Case 1 shows that for the chemical entity *D002110*, its mention of caffeine only appears in sentence 1, while the disease entity *D007674* appears in sentence 11. This chemical disease entity pair has a positive relationship, but its semantic dependence spans the entire document. So, it is tough for the model to judge the relation correctly. However, HTGRS can identify its relationship as positive, which we attribute to HTGRS fully understanding the global interaction information through entity pair-based relation reasoning. Furthermore, we confirmed this situation by re-predicting the results by removing the RS module, named w/o RS MODULE predictive label; we observe that the predictive result is wrong without RS MODULE.

However, in case 2, since the mention of chemical entity *D002220* and disease entity *D006331* co-occur in the first sentence, the model easily predicts their CID relation as a positive through semantic understanding. However, according to the BioCreative-V community annotation specification, relation instances without specific CID relation tasks do not have CID relation, so the chemical disease entity is negatively related to the real relation. This result shows that our model has certain limitations in scenarios that require background knowledge.

Table S3. Case study of the prediction results on CDR dataset. [a]

Case 1:

**PMID:** 25986755

**Document:** [1]Low functional programming of renal AT2R mediates the developmental origin of glomerulosclerosis in adult offspring induced by prenatal caffeine*(Chemical:D002110)* exposure .|[2]UNASSIGNED : Our previous study has indicated that prenatal caffeine*(Chemical:D002110)* exposure ( PCE ) could induce intrauterine growth retardation ( IUGR ) of offspring .|… .|[11]These results demonstrated that PCE could induce dysplasia of fetal kidneys*(Disease: D007674)* as well as glomerulosclerosis of adult offspring , and the low functional programming of renal AT2R might mediate the developmental origin of adult glomerulosclerosis .

**Target entity pair**: <*caffeine, dysplasia of fetal kidneys*>

**True label:** positive relationship

**w/ RS MODULE Predictive label:** positive relationship √

**w/o RS MODULE Predictive label:** negative relationship ×

Case 2:

**PMID:** 1728915

**Document:** [1]Carbamazepine*(Chemical:D002220)* - induced cardiac dysfunction*(Disease:D006331)*.|[2]Characterization of two distinct clinical syndromes .|[3]A patient with sinus bradycardia and atrioventricular block … .|[4]From the analysis of these cases , two distinct forms of carbamazepine - associated cardiac dysfunction*(Disease:D006331)* emerged … .|[7]Because carbamazepine … .

**Target entity pair**: <*Carbamazepine, cardiac dysfunction*>

**True label:** negative relationship

**w/ RS MODULE Predictive label:** positive relationship ×

**w/o RS MODULE Predictive label:** positive relationship ×

[a]Yellow highlight refers to the mention of head entity as it appears in the document, and blue highlight signifies to the mention of tail entity.

## References


[1] Po-Ting Lai and Zhiyong Lu. Bert-gt: cross-sentence n-ary relation extraction with bert and graph transformer. Bioinformatics, 36(24):5678–5685, 2020.
